# Environmental Risk Factors for Sarcoidosis

**DOI:** 10.3389/fimmu.2020.01340

**Published:** 2020-06-26

**Authors:** Marc A. Judson

**Affiliations:** Albany Medical College, Albany, NY, United States

**Keywords:** sarcoidosis, antigen, environment, infection, immunity

## Abstract

Sarcoidosis is a multisystem granulomatous disease that may affect any body organ. Sarcoidosis is associated with many environmental and occupational exposures. Because the exact immunopathogenesis of sarcoidosis is unknown, it is not known whether these exposures are truly causing sarcoidosis, rendering the immune system more susceptible to the development of sarcoidosis, exacerbating subclinical cases of sarcoidosis, or causing a granulomatous condition distinct from sarcoidosis. This manuscript outlines what is known about the immunopathogenesis of sarcoidosis and postulates mechanisms whereby these exposures could cause or exacerbate the disease. We also describe the varied environmental and occupational exposures that have been associated with sarcoidosis. This includes potential infectious exposures such as mycobacteria and Propionibacterium acnes, a skin commensal bacterium, as well as non-infectious environmental exposures including inhaled bioaerosols, metal dusts and products of combustion. Further insights concerning the relationship of environmental exposures to the development of sarcoidosis may have a major impact on the prevention and treatment of this enigmatic disease.

## Introduction

Sarcoidosis is a multisystem granulomatous disease of unknown cause. The lung is the most common organ involved with sarcoidosis at a frequency of ~90 percent (1, 2). The skin, eyes, peripheral lymph nodes and liver are also commonly involved (1, 2). Unlike sarcoidosis, the causes of many granulomatous diseases are known. Exposures that may cause granulomatous inflammation include mycobacteria and fungi that may cause granulomatous infection, (3, 4) bioaerosols including bird antigens that cause hypersensitivity pneumonitis (5) and metals including beryllium that causes chronic beryllium disease (CBD) (6). It is plausible that sarcoidosis is caused by one or several antigen exposures that initiates and possibly perpetuates the granulomatous process. Several environmental exposures have been linked to sarcoidosis. Because these exposures are disparate, they may lead to the development of sarcoidosis via different mechanisms; and in fact, it is possible that sarcoidosis represents a conglomeration of several dissimilar diseases [“the sarcoidoses” (7)]. This manuscript will explore environmental risk factors for sarcoidosis. We will briefly describe the potential role of environment antigens in leading to the granulomatous inflammation of sarcoidosis, and then focus on the available evidence supporting an association of specific environmental exposures with the development of sarcoidosis.

## Overview of Immunopathogenesis of Sarcoidosis Relative to Environmental Risk Factors

Environmental exposures are postulated to be associated with the development of sarcoidosis in four general ways. The first mechanism involves the detection and processing of antigen by antigen presenting cells such as macrophages and dendritic cells. These processed antigens are subsequently presented via human leukocyte antigen (HLA) Class II molecules to a restricted set of T-cell receptors on naive T lymphocytes that are primarily of the CD4^+^ class (8). An interplay of antigen, HLA class II molecules, and T-cell receptors occurs at the HLA molecule binding site and is thought to be essential for sarcoidosis to develop (9). These events induce a polarization of the T lymphocytes to a Th1/Th17 phenotype, (10) followed by cellular recruitment, proliferation, and differentiation leading to formation of the sarcoid granuloma. This mechanism is thought to be common across most granulomatous lung diseases known to be caused by a specific antigen, and therefore it is possible that the immune system may not be dysregulated in this instance.

There is a large body of evidence to support this proposed mechanism for the immunopathogenesis of sarcoidosis. Various HLA gene alleles have been associated with development of sarcoidosis, (11, 12) protection from developing sarcoidosis, (11, 12) and specific disease phenotypes (11, 12). Further analyses have suggested that such HLA gene polymorphisms result in conformational changes in the antigen binding pockets of HLA molecules (13). Additional evidence supporting this proposed mechanism for sarcoidosis relates to CBD, a phenotypic mimic of sarcoidosis both radiographically and pathologically, (14, 15) which is associated with specific amino acid substitutions in the HLA molecule (16, 17) Beryllium-specific oligoclonal CD4+ T lymphocytes recognize beryllium within HLA molecules with these amino acid substitutions and this recognition leads to CD4+ lymphocyte proliferation, recruitment of other T cells and monocytes to the lung, (18) and the production of Th1/Th17 cytokines that eventually results in granuloma formation (18, 19). Further indirect support of this mechanism of sarcoidosis granuloma formation is that the lung and the skin are the two most common organs involved with sarcoidosis when the disease is clinically isolated to one organ (20). The skin and the lung are particularly conductive sites for antigen capture (21) and adaptive immune responses (22). It may be that these two organs are the main “portal of entry” for antigens that elicit the sarcoidosis granulomatous response, with further organ involvement requiring dissemination of antigen and/or other inflammatory mechanisms, (20) such as T-cell homing (23).

Although there is abundant evidence supporting the aforementioned mechanism of antigen-induced granulomatous inflammation in sarcoidosis, this mechanism is inconsistent with several available clinical and epidemiologic data. First, the associations between various HLA alleles and sarcoidosis phenotypes are not universal, but rather ethnicity-specific (11, 12, 24). Second, although sarcoidosis patients with specific phenotypic features of sarcoidosis have statistically higher percentages of certain HLA alleles than sarcoidosis patients without those specific phenotypic features or the general population, a significant percentage of individuals in these latter two groups carry the allele of risk (12, 25). In addition, most of these allele-specific phenotypes explain a small minority of cases (12, 13, 25). Another criticism of this mechanism is that it is problematic to account for the systemic features of sarcoidosis. The granulomas of sarcoidosis are often found in multiple and disparate organs. It is unclear how causative sarcoidosis antigens could disseminate throughout the body.

The second mechanism by which environmental antigens may induce a granulomatous response in sarcoidosis involves dysregulation of the immune system leading to autoimmunity. Evidence is accumulating that autoimmunity may be involved in some forms of sarcoidosis (26–30). Autoimmunity in sarcoidosis may occur via molecular mimicry whereby antigens trigger inflammation leading to exposure of self-peptides (31). Immunologic similarities between the “foreign” trigger and the “self” peptide promote autoreactive T or B cells in a susceptible individual. It is possible that the initial granulomatous reaction in sarcoidosis is a direct consequence of an antigen exposure in a target organ, but that subsequent granulomatous reactions in other organs are the result of molecular mimicry. This mechanism might explain how sarcoidosis manifests as a systemic disease without the need for a putative antigen to disseminate throughout the body. The best evidence for autoimmunity has been demonstrated in Lofgren's syndrome, a self-limiting form of the sarcoidosis where independent groups have identified vimentin as a possible autoantigen using proteomic techniques on lung macrophages and homogenized spleen tissue (32–36). Molecular mimicry may also by alteration of the binding pocket of the HLA molecule causing a granulomatous reaction to self-antigens. This mechanism appears to be relevant in the case of chronic beryllium disease, (37) and may explain other associations of metal exposures to the development of sarcoidosis or sarcoidosis-like reactions (vide infra). Antinuclear antibodies have been found in more than one-quarter of sarcoidosis patients in some cohort, suggesting autoimmunity may occur in sarcoidosis and may cause overlap syndromes with connective tissue diseases (38). Another form of “autoimmunity” could occur from autophagy that has been shown to promote MHC-II (major histocompatibility complex-II) presentation of proteins from intracellular sources (39). Perhaps environmental antigens first stimulate HLA molecules that interact with intracellular proteins as the result of autophagy.

A third mechanism by environmental exposures may induce sarcoidosis is by acting as an adjuvant and/or as a non-specific stimulator/dysregulator of the immune system. Such a mechanism would not directly cause sarcoidosis but would render the immune system more susceptible to another antigen or mechanism that could cause sarcoidosis. Such a mechanism may be analogous to a drug-induced sarcoidosis (DISR) like reaction that occurs with immune checkpoint inhibitor (ICI) therapy (40). ICIs are drugs that not only enhance anti-tumor activity, but also stimulate the immune system resulting in numerous immune-related adverse events (irAEs) One of several of these irAEs is a DISR, although < 10% of irAEs were DISRs in one series (41). It is therefore plausible that ICIs are not stimulating the immune system specifically to cause sarcoidosis but enhancing the risk of sarcoidosis in susceptible individuals.

**Figure 1:**
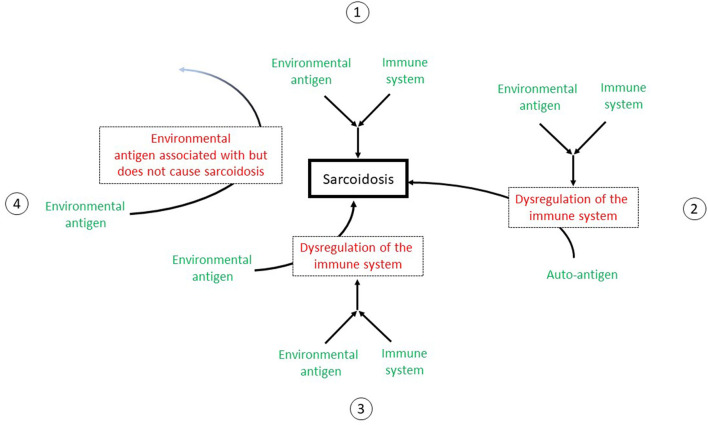
A depiction of the possible mechanisms to explain the association of environmental exposures to sarcoidosis. 1: The environmental exposure may act as an antigen to stimulate the immune system to directly cause sarcoidosis. The immune system is functioning normally with a appropriate response to the antigen. 2: The antigen interacts with the immune system to cause its deregulation. The immune system then acts abnormally to cause sarcoidosis. 3: The antigen acts as an adjuvant that acts as an adjuvant to stimulate or dysregulate the immune system but not directly cause sarcoidosis. However, the immune system is now “primed” such that another antigen or stimulus can now interact with the immune system to cause sarcoidosis. 4: The environmental exposure is a cofounder. Although this exposure is associated with sarcoidosis, it does not cause sarcoidosis.

Finally, environmental exposures that are epidemiologically associated with sarcoidosis may not be involved in any mechanism of disease development, as association does not prove causation. Figure 1 outlines the possible mechanisms to explain the association of environmental exposures to sarcoidosis.

## The Role of Genetics

The aforementioned discussion concerning the relationship between sarcoidosis and potential environmental exposures is incomplete without a discussion of the importance of genetics. It is hypothesized that a combination of genetic and environmental factors contribute to the development of sarcoidosis (42). A recent large familial aggregation study showed that heritability of the disease was 39%, (43) suggesting that genetic variation is an important contributing factor to the risk of sarcoidosis. Various HLA polymorphisms have been associated with development of sarcoidosis, protection from sarcoidosis and certain phenotypic expressions of sarcoidosis (11, 13). In addition, genome-wise association studies have reported variations numerous non-HLA genes that are associated with sarcoidosis. These include annexin A11 (44) that is involved in cell division and apoptosis, NOTCH4 (45) that regulates the activity of T cell immune responses, and BTNL2 (45, 46) that is involved in T cell activation. We suspect that many of the aforementioned mechanisms described concerning the association of environmental exposures to sarcoidosis depend on specific genetic factors. We envision that in the future, the etiology of sarcoidosis will be personalized whereby certain genetics traits present in an individual will suggest that certain specific exposures will place the subject at risk of developing sarcoidosis.

## Potential Infectious Causes of Sarcoidosis

Infectious agents have been suspected as being a possible cause of sarcoidosis. However, data supporting this conjecture are inconsistent and unconvincing. There is an abundance of indirect evidence that mycobacteria are involved in the development of sarcoidosis. Two meta-analyses of studies evaluating infectious agents as a cause of sarcoidosis have suggested an etiologic link between mycobacteria and sarcoidosis (47, 48). Molecular techniques have identified mycobacterial components in sarcoidosis tissues in some (49–51) but not all (52, 53) studies. Mycobacterial catalase-peroxidase protein (mKatG) has been identified in sarcoidosis tissues. mKatG has similar physicochemical properties to the Kveim-Siltzbach reagent that induces granulomatous inflammation almost exclusively in sarcoidosis patients (54) T-cell responses to mKatG have been demonstrated in peripheral blood monocytes of sarcoidosis patients (55, 56) with even more robust T-cell responses in bronchoalveolar lavage fluid (55, 57) and strongest responses in those with active disease (55, 57) Similar findings have not been demonstrated in other lung diseases (55, 57). It should be noted that the demonstration of mycobacterial antigens in sarcoidosis patients and granulomatous immune responses to mycobacterial antigens does not imply that sarcoidosis is a form of mycobacterial infection. Rather, it may be that some poorly degraded mycobacterial antigens contribute to the immune process of sarcoidosis without the presence of viable invasive mycobacterial organisms (48).

There is abundant evidence that Propionibacterium acnes, a skin commensal bacterium, is associated with sarcoidosis. This microorganism is the only one that has been cultured from sarcoidosis lesions (58, 59). Numerous studies have identified specific immune responses to Propionibacterium acnes in sarcoidosis patients but much less to none in non-sarcoidosis controls (52, 60, 61). Propionibacterium acnes was highly associated with sarcoidosis in a meta-analysis of studies evaluating infectious agents as a cause of the disease (47).

Numerous other infectious agents have been implicated in the immunopathogenesis of sarcoidosis. Several of these infectious agents are listed in Table 1. Implicated infectious agents include bacteria, mycobacteria, and fungi.

**Table 1:** Selected infectious agents associated with sarcoidosis^*^.

**Infectious agents**	**Immunologic**	**Molecular**	**Culture**
Mycobacterium tuberculosis	√(50, 55, 62)	√(49, 56, 63)	
Other Mycobacteria	√(57, 64)	√(49, 51)	
Propionibacterium acnes	√(52, 65, 66)	√(60)	√(58, 59)
Fungi	√(67, 68)		
Borrelia	√(69)	√(70, 71)	

* *References are in parentheses*.

Several trials of antibiotics for sarcoidosis have targeted suspected infectious pathogens. Therapy with concomitant levofloxacin, ethambutol, erythromycin and rifampin (CLEAR) has been used for the treatment of sarcoidosis by targeting presumed mycobacterial pathogens. An open label trail of CLEAR for pulmonary sarcoidosis showed an improvement in forced vital capacity in 8 of the 15 enrolled patients who were able to tolerate the study drugs for the full 8 weeks of the study (72). A subsequent small (*N* = 29) single-blind placebo-control trial of the CLEAR regimen for cutaneous sarcoidosis showed a statistically significant greater reduction in lesion size with CLEAR than placebo (73). A larger randomized double-blind placebo-controlled trial of CLEAR for pulmonary sarcoidosis is currently underway. Several case series of tetracyclines, including doxycycline and minocycline, have been reported as effective for cutaneous sarcoidosis (74, 75). These reports were unblinded descriptions of treated cases without comparisons with a control group. Furthermore, it is unclear if the mechanism of action of these tetracyclines was antibacterial or anti-inflammatory (76). Case reports and case series have shown a benefit from with clarithromycin for presumed Propionibacterium acnes infection in sarcoidosis patients, (77) as well as a benefit from empiric anti-fungal therapy (78). However, these reports also contained no control patients and/or were poorly designed to rigorously demonstrate a clinically relevant endpoint. In summary, the available evidence does not clearly demonstrate that therapy vs. a specific infectious pathogen is useful for the treatment of sarcoidosis. As previously mentioned, this does not exclude infectious organisms being involved in the immunopathogenesis of sarcoidosis, as an antigen of a microorganism may stimulate the immune system in ways that promote the granulomatous inflammation of sarcoidosis.

Besides examining individual infectious pathogens as causes of sarcoidosis, human microbiotica may have an important role in disease development. Human microbiotica regulate several physiological processes including metabolic functions and immune homeostasis (79). Alterations in the gut and respiratory microbiome have been associated with several inflammatory diseases including autoimmune diseases and cancer (80–83). It is plausible that sarcoidosis may be associated with specific changes in the composition of lung or gut microbiotica. However, a few studies examining changes in the lung microbiome have failed to identify distributions that are specific for sarcoidosis (84, 85). One recent study did suggest that Atopobium and Fusobacterium may be associated with sarcoidosis, (86) and another found that microbiota in bronchoalveolar lavage of sarcoidosis patients was less diverse and abundant compared to healthy controls (87). However, it is unclear whether these changes in the lung microbiome are causing sarcoidosis or are a result of the disease.

## Potential Non-Infectious Environmental Risk Factors for Sarcoidosis

There are numerous non-infectious environmental risk factors associated with sarcoidosis. These risk factors include working in various occupations, exposure to various substances, and dwelling in particular environments (Table 2). Most of these associations are epidemiologic. Numerous epidemiologic studies have demonstrated that sarcoidosis occurs most commonly in the Spring season (88–91). This suggests that some sarcoidosis cases may result from inhalation of an organic bioaerosol that is more abundant in the springtime, possibly analogous to summer-type hypersensitivity which is a form of hypersensitivity pneumonitis in Japan caused by inhalation of certain fungi that reach high concentrations in the summer air (113). Several other epidemiologic analyses have found that the prevalence of sarcoidosis is associated with exposure to other organic bioaerosols such as exposure to musty odors at the workplace (103) and exposure to industrial organic dusts (104, 105).

**Table 2:** Non-infectious environmental risk factors associated with sarcoidosis.

**General category**	**Type of study**	**Study population**	**Findings**	**Reference**
Space and/or time clustering: Seasonal variation in disease incidence	Space-time analysis	^(88)^- Rochester, MN;^(89)^- Turkey;^(90)^-New Zealand;^(91)^-Catlonia, Spain	Increased risk in the Spring	(88–91)
	Space-time analysis	USA Veterans	Increased risk in the Summer	(92)
	Space-time analysis	Rochester, MN	Decreased incidence in Autumn	(93)
Space clustering	Space clustering analysis	Ireland	Increased prevalence in certain regions of Ireland	(94)
	Space clustering analysis	Ireland	Higher risks in the North than South	(94)
	Space clustering analysis	Japan	Higher rates of sarcoidosis in Northern than Southern Japan	(95)
	Space clustering analysis	Hospitalized patients US military	Higher frequency in the Southeast US than other US locations	(96)
	Space clustering analysis	South Carolina	Increased prevalence near the coastline of South Carolina	(97)
	Space-time analysis vs. standard incidence and prevalence rates of sarcoidosis	Poland, living in forest of arable land	Increased incidence	(98)
	Co-inertia analysis plus linear model of hospitalized patients	Switzerland, Living near areas with metal industries	Increased prevalence	(99)
	Co-inertia analysis plus linear model of hospitalized patients	Switzerland, living in areas with potato production, artificial meadows, grain production	Increased prevalence	(99)
Occupational exposure	firefighter cohort vs. EMT cohort	NYC, Firefighters	Increased incidence and/or prevalence	(100)
	firefighter cohort vs. police cohort	Prov, RI, Firefighters	Increased incidence and/or prevalence	(101)
	Hospitalizations rates of Blacks in the US Navy	Black US Navy ship servicemen	Increased risk	(102)
	Hospitalizations rates of Blacks in the US Navy	Black US Navy Aviation structural mechanics	Increased risk	(102)
	Hospitalizations rates of Blacks in the US Navy	White US Navy ship culinary specialists	Increased risk	(102)
	Case-control US	Using insecticides	Increased risk	(103)
	Case-control US	Musty odor at work	Increased risk	(103)
	Case-control US	Building materials	Increased risk	(104)
	Case-control US	Hardware	Increased risk	(104)
	Case-control US	Garden supplies	Increased risk	(104)
	Case-control US	Mobile homes	Increased risk	(104)
	Case control US	Industrial organic dusts	Increased risk	(104)
	Case-control^*^AA Detroit, MI	Education	Increased risk	(105)
	Case-control^*^AA Detroit, MI	Metal machining	Increased risk	(105)
	Case-control^*^AA Detroit, MI	Metal working	Increased risk	(105)
	Case-control^*^AA Detroit, MI	Transportation services	Increased risk	(105)
	Incidence vs. exposure	Sweden: Silica exposure in foundry workers	Increased risk	(106)
	Longitudinal cohort of construction workers, exposed vs. unexposed to silica	Sweden, construction workers	Increased risk	(107)
	Silica in lung and lymph node biopsy, Case series: 2 cases	Silica (metal-halide lamp production)	Increased risk	(108)
	Case-control with sarcoidosis patients and their siblings who did not have sarcoidosis	AAs USA, Photocopier toner exposure	Increased risk	(109)
	Tracking sarcoidosis incidence in FDNY workers pre and post WTC disaster	NYC, World Trade Center dust exposure	Increased incidence	(110)
	Case-control^*^AA Detroit MI	AA Detroit MI, Working in high humidity	Increased risk	(105)
	Case-Control^*^AA Detroit MI	AA Detroit MI, Working with titanium	Increased risk	(105)
	Case-Control^*^AA Detroit MI	AA Detroit MI, Working with vegetable dust	Increased risk	(105)
	Elicited history of exposure and analyzed lung biopsy specimens	Man-made mineral fibers	Increased risk	(111)
Environmental exposure	Case-Control with dose response SC	SC, Wood stove use	Increased risk	(112)
	Case-Control with dose response SC	SC, Fireplace use	Increased risk	(112)
	Case-Control^*^AA Detroit MI	AA Detroit MI, Musty odors	Increased risk	(105)
	Case-control SC	SC, Non-public water use	Increased risk	(112)
	Case-control SC	SC, Living/working on a farm	Increased risk	(112)

* *Controls were unaffected siblings of sarcoidosis cases; MN, Minnesota; NYC, New York City; RI, Rhode Island; AA, African American; FDNY, Fire Department of New York City; WTC, World Trade Center; SC, South Carolina*.

Sarcoidosis is also associated with inorganic aerosol exposures, particularly with several metal dusts. This association is not surprising, as CBD from beryllium exposure is a clinical mimic of sarcoidosis. Sarcoidosis is not only associated with several occupations directly involved with manipulations of metals (102, 105) but also more subtle exposures including photocopier toner (109) that contains copper, iron, and silica (114). One report found a significant association of man-made mineral fiber exposure and the development of sarcoidosis, and then went further to perform electron microscopy quantitative analysis on previous lung specimens in the sarcoidosis group and found that half of them silica, aluminum and/or titanium (111).

Exposure to combustible products, especially combustible wood, has been associated with the development of sarcoidosis. A prototypical example of this association is the high incidence and prevalence rates sarcoidosis that is observed in firefighters (100, 101). In one analysis, emergency medical technicians (EMTs) served as a control group to the firefighters because both groups went to fire sites (100). The annual incidence rate of sarcoidosis was extremely high (44/100,000) in the firefighters whereas it was 0 in the EMTs. Wood stove use and fireplace use have also been associated with the development of sarcoidosis (112). The rigor of this association was strengthened by demonstrating a significant dose-response relationship of both wood stove and fireplace use to the frequency of sarcoidosis. Dust from the World Trade Center disaster has been associated with increased rates of developing sarcoidosis within the first 4 years after exposure (110). However, World Trade Center dust was a heterogenous exposure, and it is unclear whether the causative substance(s) was a combustible product, metal or gas.

Higher prevalence rates of sarcoidosis have been observed in Northern latitudes such as Northern Europe and Northern Japan, (95, 115) and it has been postulated that this relates to decreased sunlight exposure causing a deficiency in 1,25-dihydroxy-vitamin D (116). A deficiency in 1,25-dihydroxy-vitamin D is associated with decreased production of the antimicrobial peptide cathelicidin that contributes to the development of infectious granulomatous diseases such as tuberculosis (95, 117). A relative deficiency in 1,25-dihydroxy-vitamin D may also explain the increased frequency of sarcoidosis in Blacks, as the ability to convert 7-dehydrocholesterol to previtamin D is suppressed because of skin pigmentation (118).

Some exposures associated with sarcoidosis are problematic to explain such as working in education (105) or the culinary arts (102). This may relate to the aforementioned concept that sarcoidosis may involve an initial portal of entry where a causative antigen first interacts with the immune system and then requires additional inflammatory modulation to cause disease. In an analysis that focused on mortality from sarcoidosis and not the incidence or prevalence of disease, women with sarcoidosis were more likely to have exposure from person-to-person contact (administration and banking) whereas men who were more likely to have inhalational exposures (119). This may explain why woman are more likely to develop non-pulmonary sarcoidosis than men, (20) and it might also explain how non-respiratory exposures may be mechanistically linked to the development of sarcoidosis.

## Animal and Experimental Models of Granulomatous Disease

Various animal and experimental models of granulomatous have been developed that have involved exposure to environmental substances (120). Carbon nanotube induced granulomatous lung disease has been demonstrated in an animal model and has shown several similarities to sarcoidosis (121, 122). Numerous components of infectious agents, particularly mycobacteria and Propionibacterium acnes have mimicked features of sarcoidosis in animal models (123–126). These models have demonstrated similar immune responses in terms of T-cell function and the production of cytokines seen in sarcoidosis (120).

## Summary

In conclusion, sarcoidosis is associated with several environmental exposures including infectious agents, non-infectious organic antigens, metals, combustible products, and other inorganic substances. These disparate exposures may suggest that sarcoidosis represents a collection of different disorders that all result in the development of a multisystem granulomatous disease. Alternatively, these varied exposures may each stimulate the immune system in different ways such that a specific immune pathway that leads to sarcoidosis is promoted. This could include the induction of autoimmunity. Genetics factors are most probably an important aspect of these mechanisms. Further insights concerning the relationship of environmental exposures to the development of sarcoidosis may have a major impact on the prevention and treatment of this enigmatic disease.

## Author Contributions

The author confirms being the sole contributor of this work and has approved it for publication.

## Conflict of Interest

The author declares that the research was conducted in the absence of any commercial or financial relationships that could be construed as a potential conflict of interest.
